# MUC1 overexpression predicts worse survival in patients with non-small cell lung cancer: evidence from an updated meta-analysis

**DOI:** 10.18632/oncotarget.19861

**Published:** 2017-08-03

**Authors:** Xing Huang, Qi Sun, Chen Chen, Yi Zhang, Xin Kang, Jing-Yuan Zhang, Da-Wei Ma, Lei Xia, Lin Xu, Xin-Yu Xu, Bin-Hui Ren

**Affiliations:** ^1^ Department of Pathology, Jiangsu Cancer Hospital, Jiangsu Institute of Cancer Research, Nanjing Medical University Affiliated Cancer Hospital, Nanjing, Jiangsu, China; ^2^ Department of Cardiothoracic Surgery, Jinling Hospital, Southern Medical University, Nanjing, Jiangsu, China; ^3^ Department of Oncology, Jiangsu Cancer Hospital, Jiangsu Institute of Cancer Research, Nanjing Medical University Affiliated Cancer Hospital, Nanjing, Jiangsu, China; ^4^ Department of General Surgery, Drum Tower Hospital, Medical School of Nanjing University, Nanjing, Jiangsu, China; ^5^ Department of Thoracic Surgery, Jiangsu Cancer Hospital, Jiangsu Institute of Cancer Research, Nanjing Medical University Affiliated Cancer Hospital, Nanjing, Jiangsu, China

**Keywords:** mucin1, biomarker, NSCLC, prognosis, meta-analysis

## Abstract

**Background:**

Previous studies on the prognostic role of MUC1 expression in non-small cell lung cancer (NSCLC) remain controversial. We conducted a meta-analysis to appraise the clinicopathological and prognostic effect of MUC1 in NSCLC patients.

**Materials and Methods:**

Searches of PubMed, EMBASE and CNKI (Chinese National Knowledge Infrastructure) were conducted and relevant studies were extracted. The pooled hazard ratio (HR) or odds ratio (OR) with 95% confidence intervals (CIs) were used to estimate effects. Heterogeneity among studies and publication bias were also evaluated.

**Results:**

A total of 15 studies with 1,682 patients were included in this meta-analysis. The pooled HRs indicated that elevated MUC1 expression was associated with poorer overall survival (HR = 2.12, 95% CI: 1.47–3.05; *P* < 0.001) and progression-free survival (HR = 2.00, 95% CI: 1.53-2.62; *P* < 0.001) in patients with NSCLC. Significant associations were also found in patients treated with epidermal growth factor receptor tyrosine kinase inhibitors (EGFR-TKIs) (HR = 3.16, 95% CI: 2.21–4.52, *P* < 0.001) and with a platinum-based regimen (HR = 4.35, 95% CI: 2.45–7.72, *P* < 0.001). Additionally, MUC1 overexpression was significantly associated with performance status (OR = 2.32, 95% CI: 1.13–4.73, *P* = 0.021).

**Conclusions:**

MUC1 could be a valuable biomarker of the prognoses of NSCLC patients.

## INTRODUCTION

Lung cancer is the most common type of cancer and the leading global cause of cancer-related death [1, 2]. Non-small cell lung cancer (NSCLC) accounts for 80–85% of lung cancer cases. Although progress has been achieved in the past decades, the prognosis for NSCLC is still poor, with an estimated survival rate of only 15% at 5 years [1]. Several markers, including tumor stage, tobacco smoking [3], ki-67 expression [4], cyfra21-1 [5] and XRCC1 (X-ray repair cross-complementing protein 1) polymorphism [6] have been reported as prognostic indicators of outcomes in NSCLC patients. However, it is still difficult to predict patients’ outcomes before treatment.

Mucin-1, previously called KL-6, EMA and CA15-3, is a glycoprotein present in normal epithelial tissue and in various cancers, including NSCLC [7, 8]. Mucin1 is capable of increasing the invasive and metastatic capability of tumor cells by reducing cell–cell adhesion [9] and cell-extracellular matrix adhesion [10]. Mucin1 can also interact with the family of epidermal growth factor receptors (EGFRs) and participate in the progression of carcinogenesis [11]. Therefore, Mucin1 has been extensively studied in a variety of neoplasms, including breast [12], gastric [13] and colorectal [14]. The first report of high MUC1 expression as a valuable prognostic marker for NSCLC was presented in 1998 [15]. Subsequently, numerous studies have been performed to validate this result [15–24], but it remains controversial [25–28]. A previous meta-analysis reported the prognostic value of high MUC1 expression in NSCLC patients [29] but included relatively few studies (*n* = 4). In addition, subgroup analysis based on ethnicity, method of detection and choice of therapy was not performed. Therefore, we conducted an updated meta-analysis to reappraise the effect of MUC1 expression on the prognosis of NSCLC patients.

## RESULTS

### Characteristics of eligible studies

**Figure 1:**
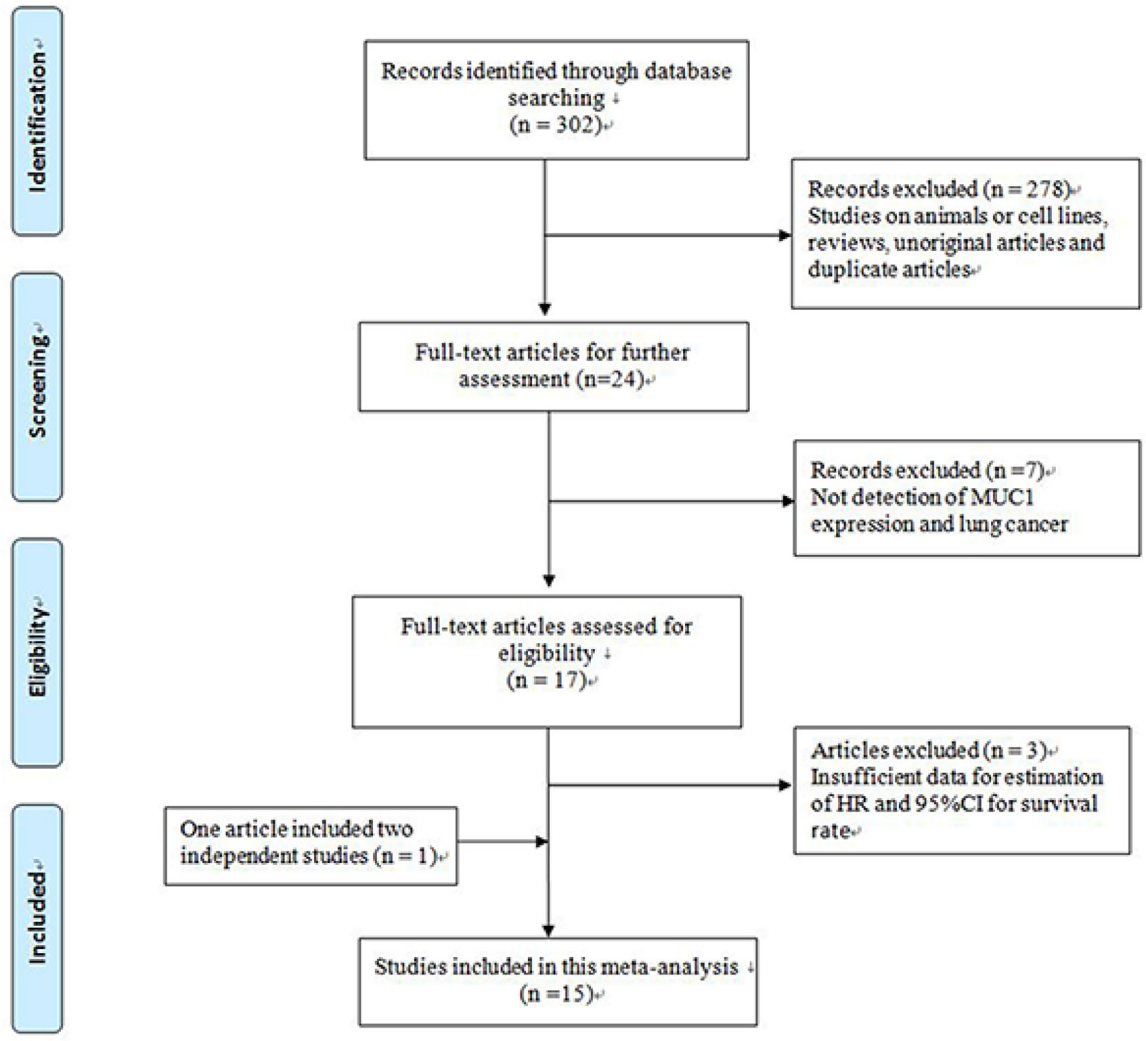
Flow chart of study selection

A total of 302 potentially relevant publications were identified after an initial search. After a review of the titles and abstracts, 278 studies were removed. Subsequently, 24 full-text articles were evaluated, seven studies were excluded for being out of scope [30–36] and another three were excluded because of insufficient data [37–39]. Miyazaki’s study included two different survival analyses separately [20], resulting in a total of 15 eligible studies containing 1,682 patients that were included in this meta-analysis [15–28] (Figure 1). Studies that reported two endpoints were analyzed separately [17–19, 21, 22].

Fourteen studies investigated the prognostic role of MUC1 on overall survival (OS), and 5 studies explored the prognostic impact of MUC1 on progression-free survival (PFS). Nine studies were from Japan, three from China, two from Germany, and one from Greece. The sample sizes ranged from 41 to 185. HRs and 95% CIs were extracted directly from the 11 studies. HRs in 4 studies were estimated by Kaplan-Meier survival curves [15, 20, 23, 27]. MUC1 expression was divided into high and low levels, and different cut-off values were selected in each study. Most studies performed experiments using the manufacturer’s instructions; some applied the median or mean levels as cut-off values, and the remaining studies defined the cut-off value independently or by using a ROC curve. Detailed characteristics of the included studies are listed in Table 1.

**Table 1:** Characteristics of studies included in this meta-analysis

Author	Year	Country	Ethnicity	Surgery	Chemotherapy	TNM	Study	HR	N	Biomarker	Method	Cutoff	High/low	Follow-up	NOS
						Stage	design	Estimate						months	score
Tomita [16]	2016	Japan	Asian	Yes	NA	I–III	R	Reported	175	OS	ELISA	500 U/mL	15/160	NA	5
Shao [25]	2014	China	Asian	No	EGFR-TKI	IIIb–IV	P	Reported	114	PFS	ELISA	332 U/mL	NA	55	7
Li [17]	2014	China	Asian	No	EGFR-TKI	IIIb–IV	P	Reported	66	OS/PFS	RT-PCR	4.2	30/36	11.2 (8.4-16.6)	7
Tanaka [18]	2012	Japan	Asian	Yes	Platinum-based	Ia–IIIa	R	Reported	103	OS/PFS	ECLIA	400 U/mL	23/80	NA	8
Situ [19]	2011	China	Asian	Yes	NA	Ib	R	Reported	178	OS/DFS	IHC	ROC	114/64	62.8 (3-157.1)	6
Miyazaki1 [20]	2010	Japan	Asian	-	NA	Ia–IV	R	SC	68	OS	ELISA	500 U/mL	50/18	NA	6
Miyazaki2 [20]	2010	Japan	Asian	-	NA	Ia–IV	R	Reported	205	OS	ELISA	500 U/mL	69/136	NA	6
Kuemmel [26]	2009	Germany	Caucasian	-	NA	Ia–IIIb	R	Reported	85	OS	IHC	IRS ≥ 3	44/41	48.1 (1.3–114.9)	7
Woenckhaus [27]	2008	Germany	Caucasian	Yes	NA	Ia–IIIb	R	SC	96	OS	IHC	5%	73/23	35	7
Ishikawa [21]	2008	Japan	Asian	No	EGFR-TKI	IIIb–IV	R	Reported	70	OS/PFS	ECLIA	500 U/mL	35/35	NA	8
Fujiwara [22]	2008	Japan	Asian	No	EGFR-TKI	IIIa–IV	R	Reported	41	OS/PFS	ECLIA	500 U/mL	22/19	20.6	8
Inata [28]	2007	Japan	Asian	-	NA	Ia–IV	R	Reported	103	OS	ELISA	NA	34/69	NA	6
Tsutsumida [23]	2004	Japan	Asian	Yes	NA	NA	R	SC	185	OS	IHC	25%	45/140	NA	6
Hirasawa [24]	2000	Japan	Asian	No	Platinum-based	IIIb–IV	R	Reported	100	OS	ELISA	32 U/mL	11/19	54	6
Guddo [15]	1998	Greece	Caucasian	Yes	NA	Ia–IIb	R	SC	93	OS	IHC	25%	40/53	62 (45-74)	6

P: prospective; R: retrospective; NA: not available; OS: overall survival; PFS: progression-free survival; DFS: disease-free survival; SC: survival curve; ECLIA: electrochemiluminescence immunoassay; ELISA: enzyme-linked immunosorbent assay; IHC: immunohistochemistry; NOS: Newcastle-Ottawa score; ROC: receiver operating characteristic.

### Results

### MUC1 and OS

**Figure 2:**
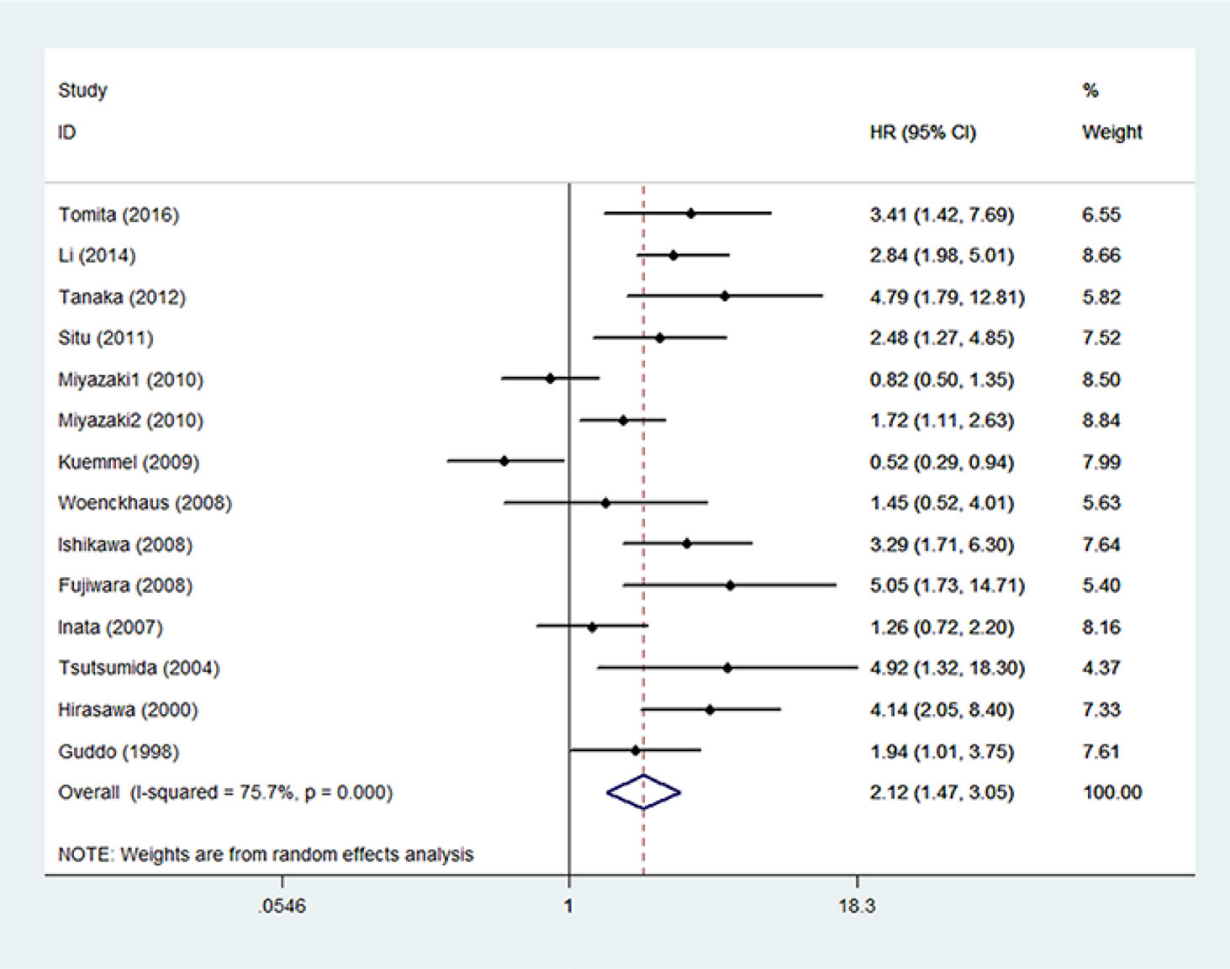
The correlation between MUC1 expression and overall survival in NSCLC patients

Fourteen studies involving 1,568 patients investigated the association between MUC1 and OS [15–24, 26–28]. The pooled HR was 2.12 (95% CI: 1.47–3.05; *P* < 0.001) (Figure 2), indicating that elevated MUC1 expression was significantly associated with poor OS. As heterogeneity was significant, a random-effects model was used (I^2^ = 75.7%; *P* < 0.001). To detect potential heterogeneity, we conducted subgroup analysis by ethnicity, surgical intervention, chemotherapy regions, sample type, sample size and cut-off value (Table 2). Subgroup analysis according to ethnicity indicated that elevated MUC1 expression had a significantly prognostic value in Asian populations (HR = 2.49; 95% CI = 1.73–3.59; *P* < 0.001). In the subgroup analysis by sample type, a significantly worse OS was detected in the sera group (HR = 2.38; 95% CI = 1.47–3.82; *P* < 0.001). When we conducted subgroup analysis by chemotherapy regions, a significant association was found in the EGFR-TKIs subgroup (HR = 3.16, 95% CI: 2.21–4.52, *P* < 0.001) and in the platinum-based regimen subgroup (HR = 4.35, 95% CI: 2.45–7.72, *P* < 0.001). Subgroup analyses suggested that elevated MUC1 expression predicted poor OS in patients with NSCLC, regardless of the sample size (< 100 and ≥ 100) and status of surgical intervention (Yes and No).

**Table 2:** Meta-analysis results

	No. of studies	No. of patients	HR (95% CIs)	Model	Heterogeneity test		
					*Q*	I-squared	*P*-value
**OS**							
**Overall**	14	1568	2.12 (1.47,3.05)	Random	53.48	75.70%	< 0.001
**Surgical intervention**							
Surgery	6	830	2.61 (1.85,3.68)	Fixed	4.82	0.00%	0.438
Non-surgery	4	277	3.34 (2.43,4.60)	Fixed	1.39	0.00%	0.707
**Chemotherapy**							
EGFR-TKI	3	177	3.16 (2.21,4.52)	Fixed	0.95	0.00%	0.622
Platinum-based	2	111	4.35 (2.45,7.72)	Fixed	0.06	0.00%	0.814
**Ethnicity**							
Asian	11	1294	2.49 (1.73,3.59)	Random	33.15	69.80%	< 0.001
Caucasian	3	274	1.10 (0.45,2.73)	Random	9.18	78.20%	0.01
**Sample type**							
Sera	8	865	2.38 (1.47,3.82)	Random	29.11	76.00%	< 0.001
Tissue	6	703	1.82 (0.97,3.44)	Random	24.15	79.30%	< 0.001
Sample size							
Large	7	1049	2.56 (1.72,3.82)	Random	13.12	54.30%	0.041
Small	7	519	1.71 (0.94,3.14)	Random	36.32	83.50%	< 0.001
**Cutoff value**							
500 U/ml	5	559	2.20 (1.19,4.10)	Random	18.71	78.60%	0.001
**PFS**							
**Overall**	5	394	2.00 (1.53,2.62)	Fixed	6.04	33.80%	0.196

OS: overall survival; HR: hazard ratio; CI: confidence interval; OS: overall survival; PFS: progression-free survival.

### MUC1 and PFS

**Figure 3:**
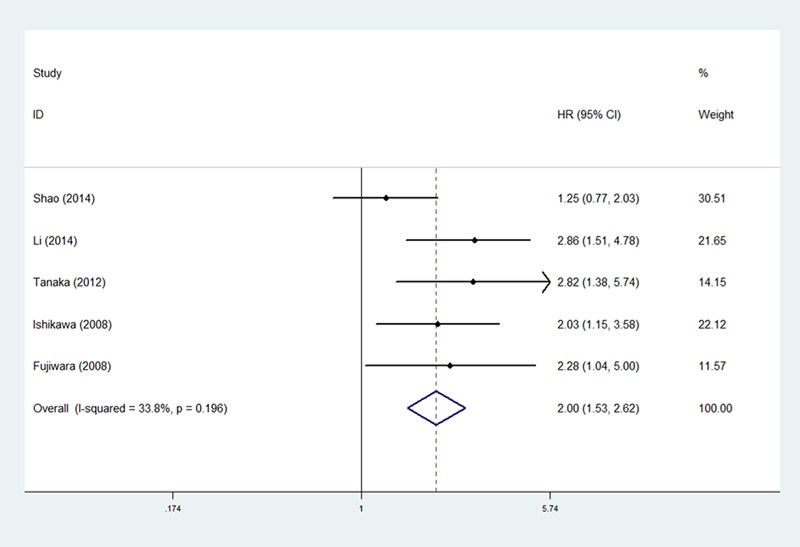
The correlation between MUC1 expression and progression-free survival in NSCLC patients

Five studies comprising 394 patients evaluated the association between MUC1 expression and PFS [17, 18, 21, 22, 25]. The results indicated that high MUC1 expression was associated with poor PFS (HR = 2.00, 95% CI: 1.53–2.62, *P* < 0.001) (Figure 3), without significant heterogeneity (I^2^ = 33.80%, *P* = 0.196).

### MUC1 and clinicopathological parameters

Eight studies examined the relevance between MUC1 expression and the clinical features of NSCLC [16–19, 21, 22, 24, 27]. Pooled data revealed that elevated MUC1 expression was significantly related to performance status (≥ 2 vs. < 2; OR = 2.32, 95% CI: 1.13–4.73, *P* = 0.021). However, no significant association was found with gender (male vs. female), age (≥ 65 vs. < 65), smoking history (yes vs. no), tumor size (> 3 cm vs. ≤ 3 cm), histology (AD vs. no-AD), and lymph node metastasis (yes vs. no). Some clinical features such as differentiation, TNM stage and distant metastasis were not included in our analysis due to a lack of data. The details of our analysis are shown in Table 3.

**Table 3:** Meta-analysis of the association between MUC1 and the clinicopathological features of NSCLC

Characteristics	No. of studies	No. of patients	OR (95% CI)	*P*	Heterogeneity	
					I^2^	P_h_
**Gender (male vs. female)**	8	829	1.32 (0.92,1.89)	0.13	17.70%	0.29
**Age (≥ 65 vs. < 65)**	3	456	1.72 (0.65,4.58)	0.277	54.90%	0.109
**Smoking history (yes vs. no)**	4	385	1.47 (0.88,2.45)	0.143	44.90%	0.142
**Tumor size (> 3 cm vs. < 3 cm)**	3	374	1.00 (0.54,1.86)	0.993	19.10%	0.29
**Histology (AD vs. no-AD)**	8	829	1.25 (0.52,3.02)	0.618	77.50%	< 0.001
**Lymph node metastasis (yes vs. no)**	3	374	1.24 (0.64,2.41)	0.53	31.50%	0.232
**Performance status (≥ 2 vs. < 2)**	3	177	2.32 (1.13,4.73)	0.021	0.00%	0.435

OR: odds ratio; CI: confidence interval; AD: adenocarcinoma; P_h_: P_heterogeneity._

### Publication bias

**Figure 4:**
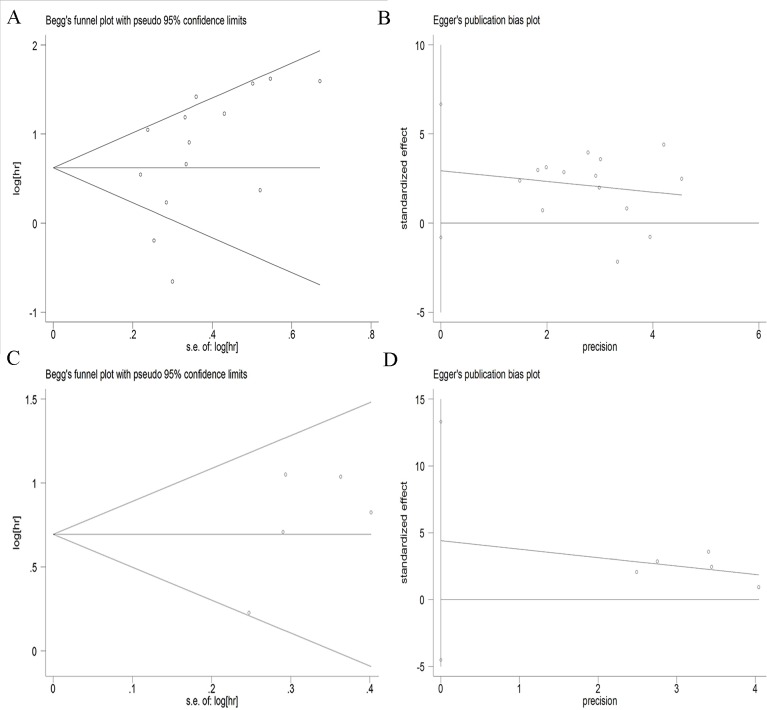
Begg’s funnel plots and Egger’s linear plots for the studies involved in the meta-analysis (**A**) Begg’s funnel plot for overall survival; (**B**) Egger’s linear plot for overall survival; (**C**) Begg’s funnel plot for progression-free survival; (**D**) Egger’s linear plot for progression-free survival.

Begg’s funnel plot and the Egger’s linear regression test were conducted to evaluate publication bias in the literature. No significant publication bias was detected by both Begg’s test (*P* = 0.208 for OS and *P* = 0.327 for PFS) and the Egger’s test (*P* = 0.604 for OS and *P* = 0.514 for PFS) (Figure 4). Therefore, no evidence of publication bias was noted.

### Sensitive analysis

**Figure 5:**
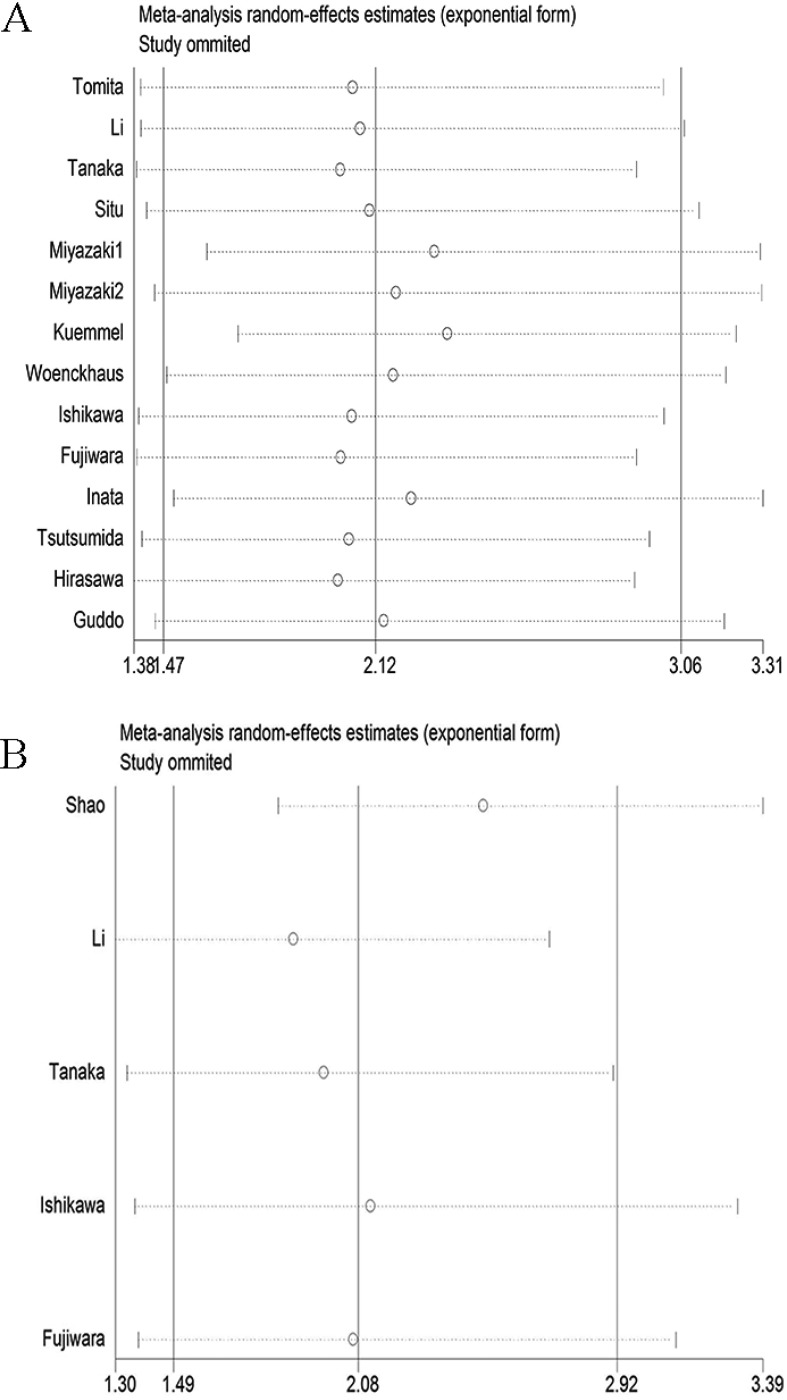
Sensitivity analysis of the meta-analysis (**A**) overall survival; (**B**) progression-free survival.

We adopted the “leave-one-out” scheme (i.e., the analysis is conducted using all studies except one) to explore the influence of individual studies on the pooled HRs. The results showed that the pooled HRs were not materially altered, which suggested that no individual study significantly affected the pooled results (Figure 5).

## DISCUSSION

To the best of our knowledge, only one meta-analysis on the prognostic value of MUC1 expression in NSCLC had previously been performed [29]. Our meta-analysis included three times more patients than the previous study, and the studies included in our analysis used more detailed information and patients with longer follow-up intervals. As a result, we were able to obtain more relevant results.

Our meta-analysis combined the results from 15 individual studies with 1,682 NSCLC patients and found that MUC1 overexpression had significantly prognostic value for OS (HR = 2.12, 95% CI: 1.47–3.05; *P* < 0.001) and PFS (HR = 2.00, 95% CI: 1.53–2.62, *P* < 0.001) in NSCLC patients. This link was observed in both surgical and non-surgical treatment groups. Subgroup analysis by ethnicity indicated the result was significant for the Asian subgroup (HR = 2.49, 95% CI: 1.73–3.59, *P* < 0.001), but not for the Caucasian subgroup (HR = 1.10, 95% CI: 0.45–2.73, *P* = 0.832). Considering the limited number of Caucasian patients in our analysis, more studies should be conducted. When stratified by sample type, a significant risk was found in the sera group (HR=2.38, 95% CI: 1.47–3.82, *P* < 0.001), indicating that MUC1 may be a convenient tumor marker for use in clinical practice. We found that 500 U/ml is the most frequently used cut-off value and is associated with significant risk (HR = 2.20, 95% CI: 1.19–4.10, *P* = 0.012).

We verified the poor prognostic role of high MUC1 expression in patients treated with a platinum-based regimen (HR=4.35, 95% CI: 2.45–7.72, *P* < 0.001) or EGFR-TKIs (HR = 3.16, 95% CI: 2.21–4.52, *P* < 0.001). Platinum-based chemotherapy has been widely adopted for the treatment of NSCLC patients and significantly improves survival and quality of life [40]. However, its efficacy varies among individuals [41]. The prognostic or predictive roles of a series of tumor markers were reported in NSCLC patients treated with platinum-based chemotherapy [42, 43], but until now, none was recommended for clinical practice. Based on our results, MUC1 might be a promising biomarker. EGFR-TKI therapy significantly improves the survival of NSCLC patients who harbor an EGFR mutation [44]. Unfortunately, there is no indicator that predicts the efficacy of EGFR-TKI therapy. Our findings indicate that MUC1 may be such an indicator, but as the sample size of our analysis is limited, large-scale prospective studies are needed to further confirm our results.

There are some limitations to our meta-analysis. First, the heterogeneity was moderately significant in the pooled HRs of OS (I^2^ = 75.7%, *P* < 0.001). Although we performed subgroup analysis and sensitivity analysis to find the source of heterogeneity, none could completely explain it. Second, this meta-analysis was limited to articles published in English or Chinese, indicating that language bias likely existed. Third, most of the studies selected were conducted on Asian populations; thus, standardized analyses should be used to apply our results to other populations. Fourth, several HRs were extracted from Kaplan-Meier curves, which might have biased our results. Finally, NSCLC consists of several subtypes, such as adenocarcinoma, squamous cell carcinoma and others. The prognosis and selection of therapy for each type are dissimilar, but detailed information on NSCLC subtypes was lacking, and we did not conduct subgroup analysis by subtypes. More studies on the association between MUC1 and NSCLC subtypes are needed.

In conclusion, our results indicate that high MUC1 expression may be a marker of poor prognosis in NSCLC patients and a promising therapeutic target. Large, well-designed prospective studies are needed to confirm our findings.

## MATERIALS AND METHODS

### Search strategy

We performed a literature search in PubMed, EMBASE, and CNKI (Chinese National Knowledge Infrastructure) databases using the following keywords: “MUC1”, “Mucin1”, “CA15-3”, “CD227”, “KL-6”, “non-small cell lung cancer”, “NSCLC”, “prognosis”, “survival”, and “outcome”. The most recent article found was published on January 13, 2017. The references of all publications and reviews were also manually searched to identify relevant studies.

### Inclusion and exclusion criteria

All included studies had to meet the following criteria: (1) evaluation of the association between MUC1 expression and NSCLC prognosis; (2) case-control studies; (3) sufficient data for estimating the hazard ratio (HR) with a 95% confidence interval (CI). The major reasons for exclusion were (1) duplicate studies; (2) case reports, comments or review articles; (3) studies lacking detailed data.

### Data extraction

Two investigators (XH and QS) performed searches and identified articles independently using a standard approach [45]. The following information was extracted: first author, publication year, nationality, ethnicity, quantitative method, cut-off value, follow-up months, hazard ratios (HR) with corresponding 95% confidence intervals (CI) for overall survival (OS) or progression-free survival (PFS) and the total number of participants, respectively. In case of discrepancies, another investigator (CC) was invited to check and discuss the original data until a consensus was reached. Quality assessment for each study included in the final analysis was performed by the same two reviewers according to the Newcastle-Ottawa quality assessment scale (NOS) [46]. NOS scores ranged from 0 to 9, and a score ≥ 6 indicates good quality in the present study.

### Statistical analysis

The intensity of the relationship between MUC1 expression and survival was expressed as HRs, and the strength of the association between MUC1 and clinical parameters was expressed as an odds ratio (OR). In some studies, HR and the 95% CI were directly obtained using univariate or multivariate survival analysis. Otherwise, a method reported by Tierney was used to reconstruct the HR and its variance from Kaplan–Meier survival curves [47]. Heterogeneity among eligible studies was estimated using a Chi-square-based *Q* test and considered statistically significant when I^2^ > 50% or *P* < 0.1 [48]. A fixed effects model (Mantel-Haenszel method) was used if there was no significant heterogeneity; otherwise, a random effects model (Der Simonian and Laird method) was used [49]. Publication bias was evaluated using Egger’s test and Begg’s test, and *P* < 0.05 was considered significant [50]. All statistical tests were conducted with STATA software version 12.0 (STATA Corporation, College Station, TX, USA) and *P* < 0.05 was considered significant.

## References

[1] Siegel RL, Miller KD, Jemal A (2017). Cancer Statistics, 2017. CA Cancer J Clin.

[2] Chen W, Zheng R, Baade PD, Zhang S, Zeng H, Bray F, Jemal A, Yu XQ, He J (2016). Cancer statistics in China, 2015. CA Cancer J Clin.

[3] Fontham ET, Correa P, Reynolds P, Wu-Williams A, Buffler PA, Greenberg RS, Chen VW, Alterman T, Boyd P, Austin DF (1994). Environmental tobacco smoke and lung cancer in nonsmoking women. A multicenter study. JAMA.

[4] Martin B, Paesmans M, Mascaux C, Berghmans T, Lothaire P, Meert AP, Lafitte JJ, Sculier JP (2004). Ki-67 expression and patients survival in lung cancer: systematic review of the literature with meta-analysis. Br J Cancer.

[5] Xu Y, Xu L, Qiu M, Wang J, Zhou Q, Xu L, Wang J, Yin R (2015). Prognostic value of serum cytokeratin 19 fragments (Cyfra 21-1) in patients with non-small cell lung cancer. Sci Rep.

[6] Chen S, Tang D, Xue K, Xu L, Ma G, Hsu Y, Cho SS (2002). DNA repair gene XRCC1 and XPD polymorphisms and risk of lung cancer in a Chinese population. Carcinogenesis.

[7] von Mensdorff-Pouilly S, Snijdewint FG, Verstraeten AA, Verheijen RH, Kenemans P (2000). Human MUC1 mucin: a multifaceted glycoprotein. Int J Biol Markers.

[8] Khodarev NN, Pitroda SP, Beckett MA, MacDermed DM, Huang L, Kufe DW, Weichselbaum RR (2009). MUC1-induced transcriptional programs associated with tumorigenesis predict outcome in breast and lung cancer. Cancer Res.

[9] Wesseling J, van der Valk SW, Hilkens J (1996). A mechanism for inhibition of E-cadherin-mediated cell-cell adhesion by the membrane-associated mucin episialin/MUC1. Mol Biol Cell.

[10] Wesseling J, van der Valk SW, Vos HL, Sonnenberg A, Hilkens J (1995). Episialin (MUC1) overexpression inhibits integrin-mediated cell adhesion to extracellular matrix components. J Cell Biol.

[11] Ren J, Li Y, Kufe D (2002). Protein kinase C delta regulates function of the DF3/MUC1 carcinoma antigen in beta-catenin signaling. J Biol Chem.

[12] Khodarev N, Ahmad R, Rajabi H, Pitroda S, Kufe T, McClary C, Joshi MD, MacDermed D, Weichselbaum R, Kufe D (2010). Cooperativity of the MUC1 oncoprotein and STAT1 pathway in poor prognosis human breast cancer. Oncogene.

[13] Wang RQ, Fang DC (2003). Alterations of MUC1 and MUC3 expression in gastric carcinoma: relevance to patient clinicopathological features. J Clin Pathol.

[14] Baldus SE, Monig SP, Huxel S, Landsberg S, Hanisch FG, Engelmann K, Schneider PM, Thiele J, Holscher AH, Dienes HP (2004). MUC1 and nuclear beta-catenin are coexpressed at the invasion front of colorectal carcinomas and are both correlated with tumor prognosis. Clin Cancer Res.

[15] Guddo F, Giatromanolaki A, Koukourakis MI, Reina C, Vignola AM, Chlouverakis G, Hilkens J, Gatter KC, Harris AL, Bonsignore G (1998). MUC1 (episialin) expression in non-small cell lung cancer is independent of EGFR and c-erbB-2 expression and correlates with poor survival in node positive patients. J Clin Pathol.

[16] Tomita M, Ayabe T, Chosa E, Nose N, Nakamura K (2016). Prognostic significance of preoperative serum Krebs von den Lungen-6 level in non-small cell lung cancer. Gen Thorac Cardiovasc Surg.

[17] Li J, Hu YM, Du YJ, Zhu LR, Qian H, Wu Y, Shi WL (2014). Expressions of MUC1 and vascular endothelial growth factor mRNA in blood are biomarkers for predicting efficacy of gefitinib treatment in non-small cell lung cancer. BMC Cancer.

[18] Tanaka S, Hattori N, Ishikawa N, Shoda H, Takano A, Nishino R, Okada M, Arihiro K, Inai K, Hamada H, Yokoyama A, Kohno N (2012). Krebs von den Lungen-6 (KL-6) is a prognostic biomarker in patients with surgically resected nonsmall cell lung cancer. Int J Cancer.

[19] Situ D, Wang J, Ma Y, Zhu Z, Hu Y, Long H, Rong T (2011). Expression and prognostic relevance of MUC1 in stage IB non-small cell lung cancer. Med Oncol.

[20] Miyazaki K, Kurishima K, Kagohashi K, Kawaguchi M, Ishikawa H, Satoh H, Hizawa N (2010). Serum KL-6 levels in lung cancer patients with or without interstitial lung disease. J Clin Lab Anal.

[21] Ishikawa N, Hattori N, Yokoyama A, Tanaka S, Nishinox R, Yoshioka K, Ohshimo S, Fujitaka K, Ohnishi H, Hamada H, Arihiro K, Kohno N (2008). Usefulness of monitoring the circulating Krebs von den Lungen-6 levels to predict the clinical outcome of patients with advanced nonsmall cell lung cancer treated with epidermal growth factor receptor tyrosine kinase inhibitors. Int J Cancer.

[22] Fujiwara Y, Kiura K, Toyooka S, Hotta K, Tabata M, Takigawa N, Soh J, Tanimoto Y, Kanehiro A, Kato K, Date H, Tanimoto M (2008). Elevated serum level of sialylated glycoprotein KL-6 predicts a poor prognosis in patients with non-small cell lung cancer treated with gefitinib. Lung Cancer.

[23] Tsutsumida H, Goto M, Kitajima S, Kubota I, Hirotsu Y, Yonezawa S (2004). Combined status of MUC1 mucin and surfactant apoprotein A expression can predict the outcome of patients with small-size lung adenocarcinoma. Histopathology.

[24] Hirasawa Y, Kohno N, Yokoyama A, Kondo K, Hiwada K, Miyake M (2000). Natural autoantibody to MUC1 is a prognostic indicator for non-small cell lung cancer. Am J Respir Crit Care Med.

[25] Shao L, Hong W, Zheng L, He C, Zhang B, Xie F, Song Z, Lou G, Zhang Y (2014). Joint serum tumor markers serve as survival predictive model of erlotinib in the treatment of recurrent non-small cell lung cancer. [Article in Chinese]. Zhongguo Fei Ai Za Zhi.

[26] Kuemmel A, Single K, Bittinger F, Faldum A, Schmidt LH, Sebastian M, Micke P, Taube C, Buhl R, Wiewrodt R (2009). TA-MUC1 epitope in non-small cell lung cancer. Lung Cancer.

[27] Woenckhaus M, Merk J, Stoehr R, Schaeper F, Gaumann A, Wiebe K, Hartmann A, Hofstaedter F, Dietmaier W (2008). Prognostic value of FHIT, CTNNB1, and MUC1 expression in non-small cell lung cancer. Hum Pathol.

[28] Inata J, Hattori N, Yokoyama A, Ohshimo S, Doi M, Ishikawa N, Hamada H, Kohno N (2007). Circulating KL-6/MUC1 mucin carrying sialyl Lewisa oligosaccharide is an independent prognostic factor in patients with lung adenocarcinoma. Int J Cancer.

[29] Xu F, Liu F, Zhao H, An G, Feng G (2015). Prognostic Significance of Mucin Antigen MUC1 in Various Human Epithelial Cancers: A Meta-Analysis. Medicine (Baltimore).

[30] Nagai S, Takenaka K, Sonobe M, Ogawa E, Wada H, Tanaka F (2006). A novel classification of MUC1 expression is correlated with tumor differentiation and postoperative prognosis in non-small cell lung cancer. J Thorac Oncol.

[31] Lappi-Blanco E, Makinen JM, Lehtonen S, Karvonen H, Sormunen R, Laitakari K, Johnson S, Makitaro R, Bloigu R, Kaarteenaho R (2016). Mucin-1 correlates with survival, smoking status, and growth patterns in lung adenocarcinoma. Tumour Biol.

[32] Blel A, Kourda N, Baltagi Ben Jilani S, Zermani R (2008). Prognostic value of morphologic subdivision of papillary renal cell carcinoma and MUC1 expression. [Article in French]. Prog Urol.

[33] Ohara G, Kurishima K, Ishikawa H, Satoh H, Hizawa N (2008). KL-6 and poor prognosis in NSCLC patients treated with gefitinib. Lung Cancer.

[34] Kaira K, Nakagawa K, Ohde Y, Okumura T, Takahashi T, Murakami H, Endo M, Kondo H, Nakajima T, Yamamoto N (2012). Depolarized MUC1 expression is closely associated with hypoxic markers and poor outcome in resected non-small cell lung cancer. Int J Surg Pathol.

[35] Zhu WF, Li J, Yu LC, Wu Y, Tang XP, Hu YM, Chen YC (2014). Prognostic value of EpCAM/MUC1 mRNA-positive cells in non-small cell lung cancer patients. Tumour Biol.

[36] Mitsuta K, Yokoyama A, Kondo K, Nakajima M, Arita K, Kohno N (2005). Polymorphism of the MUC1 mucin gene is associated with susceptibility to lung adenocarcinoma and poor prognosis. Oncol Rep.

[37] Feng J, Zhang X, Zhu H, Wang X, Ni S, Huang J (2012). FoxQ1 overexpression influences poor prognosis in non-small cell lung cancer, associates with the phenomenon of EMT. PLoS One.

[38] Giatromanolaki A, Koukourakis MI, Sivridis E, O'Byrne K, Cox G, Thorpe PE, Gatter KC, Harris AL (2000). Coexpression of MUC1 glycoprotein with multiple angiogenic factors in non-small cell lung cancer suggests coactivation of angiogenic and migration pathways. Clin Cancer Res.

[39] Ohgami A, Tsuda T, Osaki T, Mitsudomi T, Morimoto Y, Higashi T, Yasumoto K (1999). MUC1 mucin mRNA expression in stage I lung adenocarcinoma and its association with early recurrence. Ann Thorac Surg.

[40] D'Addario G, Pintilie M, Leighl NB, Feld R, Cerny T, Shepherd FA (2005). Platinum-based versus non-platinum-based chemotherapy in advanced non-small-cell lung cancer: a meta-analysis of the published literature. J Clin Oncol.

[41] Bahl A, Falk S (2001). Meta-analysis of single agents in the chemotherapy of NSCLC: what do we want to know?. Br J Cancer.

[42] Zang J, Hu Y, Xu X, Ni J, Yan D, Liu S, He J, Xue J, Wu J, Feng J (2017). Elevated serum levels of vascular endothelial growth factor predict a poor prognosis of platinum-based chemotherapy in non-small cell lung cancer. Onco Targets Ther.

[43] Peng Y, Li Z, Zhang S, Xiong Y, Cun Y, Qian C, Li M, Ren T, Xia L, Cheng Y, Wang D (2014). Association of DNA base excision repair genes (OGG1, APE1 and XRCC1) polymorphisms with outcome to platinum-based chemotherapy in advanced nonsmall-cell lung cancer patients. Int J Cancer.

[44] Janne PA, Ou SH, Kim DW, Oxnard GR, Martins R, Kris MG, Dunphy F, Nishio M, O'Connell J, Paweletz C, Taylor I, Zhang H, Goldberg Z (2014). Dacomitinib as first-line treatment in patients with clinically or molecularly selected advanced non-small-cell lung cancer: a multicentre, open-label, phase 2 trial. Lancet Oncol.

[45] Moher D, Liberati A, Tetzlaff J, Altman DG, Group P (2009). Preferred reporting items for systematic reviews and meta-analyses: the PRISMA statement. Ann Intern Med.

[46] Stang A (2010). Critical evaluation of the Newcastle-Ottawa scale for the assessment of the quality of nonrandomized studies in meta-analyses. Eur J Epidemiol.

[47] Tierney JF, Stewart LA, Ghersi D, Burdett S, Sydes MR (2007). Practical methods for incorporating summary time-to-event data into meta-analysis. Trials.

[48] Lau J, Ioannidis JP, Schmid CH (1997). Quantitative synthesis in systematic reviews. Ann Intern Med.

[49] DerSimonian R, Laird N (2015). Meta-analysis in clinical trials revisited. Contemp Clin Trials.

[50] Egger M, Davey Smith G, Schneider M, Minder C (1997). Bias in meta-analysis detected by a simple, graphical test. BMJ.

